# Notes and News

**Published:** 1906-04-21

**Authors:** 


### Notes and News.

Mr. Robinson, the Secretary of the Sunderland Infirmary, has received an intimation that the family of the late Mr. Frederick Gordon will contribute £5,000 to the building fund of the Children's Hospital at Sunderland.

The executors of the late Mrs. M. A. Irwin, to whom the testatrix left the ultimate residue of her estate for the benefit of a nursing institution and a convalescent home, to be selected by them after due inquiry, have paid over to the Metropolitan Convalescent Institution the sum of £2,316 19s., being one moiety of the residue.

In the Army Orders for April it is signified that allowances for the local purchase of additional articles of equipment and decorative articles for hospital wards will be made. These allowances will not be issued in cash, but will be expended in payment of bills incurred by medical officers in charge of hospitals containing not less than a hundred beds.

The late Mr. Frederick John Horniman, who died on March 5 last, left a sum of £1,000 each to the London Hospital, the Sussex County Hospital, the Croydon General Hospital, St. Mary's Hospital, Paddington, the Convalescent Home and Hospital at Lower Sydenham, the General Hospital at Truro, the South Devon and East Cornwall Hospital at Plymouth, and the Surgical Aid Society.

A General Meeting of the International Medical Association for the Suppression of War was held on March 21 in Paris under the presidency of Dr. J. Rivière. A number of resolutions were passed advocating an international tribune of arbitration, the substitution of a generous and humane spirit in place of racial, religious, and class hatred, and many other proposals of a pacific and humane character.

The death is announced of Deputy Surgeon-General John Tullock, late of the Army Medical Staff, who retired in 1888, after 34 years' service. He was educated at King's College, Aberdeen, where he took the degree of M.D. He joined the Army as an assistant surgeon, and in that rank was at the suppression of the Indian Mutiny at Benares, the advance to Lucknow, and several other actions, including the siege and capture of that city, the Relief of Azimghur, and the operations near Jugdespore.

The question of the reinterment of the skull of Sir Thomas Brown—which has long been preserved at the Norfolk and Norwich Hospital—was discussed at the annual meeting of the Governors on Saturday. Eventually they agreed to a proposal that the skull be returned to the tomb in the Church of St. Peter Mancroft, on condition that the latter shall be opened in the presence of representatives of the hospital with a view of satisfying them that the remains of the author of "Religio Medici" in the grave are without a skull.

In their annual report to the Board of delegates of the Hospital Saturday Fund the Distribution Committee state that during 1905 they issued 38,511 letters of recommendation to the participating medical charities, including 1,549 convalescent home letters, of which 735 were on the part-payment system, the patients and their friends contributing £1,130. The fourteen beds reserved in various institutions for consumptive patients were fully occupied, and in consequence of the insufficient accommodation many patients had to wait several weeks for admission.

Lord Ludlow, Treasurer of St. Bartholomew's Hospital, acknowledges the receipt of £2,000 as a result of the recent appeal to the Stock Exchange on behalf of the re-building fund.

Speaking on Monday at the Conference of the Friends' Social Union, at Birmingham, Dr. Hall, of Leeds, stated that an attempt he made to feed slum children on vegetarian lines failed. He deprecated the employment of married women in factories, and mentioned that of ten thousand children born in Preston, Blackburn, and Burnley, where many were largely employed in factories, 220 died before their first birthday. He had examined 2,000 Jewish children born and bred in the poorest districts of Leeds, and yet they were taller, they weighed more, and their bones and teeth were better than those of children in better-class workmen's districts; the reason being that they were better and more abundantly fed.

The Scottish Local Government Board have issued a circular to the governors of poor-houses, stating that they have had under consideration the suggestion of the Departmental Committee on Poor-law Medical Relief, etc., that, in certain poor-houses, it might be desirable to provide for the observation and treatment of persons showing symptoms of mental disorder believed to be temporary and not to necessitate removal to an asylum. The Board think it right to bring this matter specially under the notice of house committees, and to assist them in arriving at a decision, should they be disposed to favour the recommendation of the Departmental Committee, a note of the conditions under which the Board are prepared to sanction the reception of such cases in a poor-house is enclosed. These conditions provide in detail for the treatment of the patients on hospital lines.

